# Neuromodulation for Pain and Fatigue in Hereditary and Acquired Neuromuscular Conditions: A Narrative Review

**DOI:** 10.1007/s11916-026-01556-7

**Published:** 2026-09-03

**Authors:** Mert Sabiroglu, William David Arnold

**Affiliations:** 1 School of Medicine, Koc University, Istanbul, Turkey; 2 Department of Physical Medicine and Rehabilitation, University of Missouri, Columbia, MO USA

## Abstract

**Introduction:**

Pain and fatigue are highly prevalent in neuromuscular (NM) diseases, including motor neuron, peripheral nerve, junctional, and myopathic pathologies. Their multifactorial pain mechanisms as neuropathic, nociceptive, and mixed often remain resistant to conventional pharmacologic and rehabilitative strategies. The long-term use of opioids carries significant adverse effects. Given these limitations, neuromodulation offers a non-pharmacologic approach to address pain and functional impairment in neuromuscular diseases.

**Objective:**

This narrative review synthesizes existing evidence and biological rationale for neuromodulation in NM disorders, summarizing prior and ongoing research and outlining future directions for clinical applications.

**Methods:**

A targeted literature search was performed across PubMed, and ClinicalTrials.gov through October 2025 for human studies using invasive or non-invasive neuromodulation. transcranial direct-current stimulation (tDCS), repetitive transcranial magnetic stimulation (rTMS), spinal cord stimulation (SCS), dorsal root ganglion (DRG) stimulation, peripheral nerve stimulation (PNS), vagus nerve stimulation (VNS), and trigeminal nerve stimulation (TNS) are searched for NM diseases. Eligible studies involved patients with NM diseases reporting pain, fatigue, or functional outcomes. Data were qualitatively synthesized by disease category and stimulation modality, and ongoing clinical trials were summarized.

**Results:**

The available literature supports a biologically plausible and clinically promising role for neuromodulation in selected neuromuscular pain phenotypes, particularly where neuropathic mechanisms predominate. However, the evidence base remains fragmented, with limited disease specific trials and substantial heterogeneity in patient populations, stimulation parameters, and reported endpoints. These limitations currently prevent definitive conclusions about efficacy or routine clinical applicability.

**Conclusions:**

Neuromodulation offers an alternative, non-drug option to help relieve pain and fatigue in people with neuromuscular disorders. By targeting maladaptive excitability, central sensitization, and neuro-immune pathways, it holds potential to complement disease-modifying and rehabilitative strategies. However, evidence remains preliminary; standardized multicenter trials, biomarker-guided stratification, and integrative approaches are needed to define efficacy and optimize clinical translation.

## Introduction and Standard of Care in Pain Management for Neuromuscular Disorders

Neuromuscular diseases are defined not only by muscle weakness and atrophy, but also by chronic pain that can affect quality of life. This pain is often multifactorial. In some patients, it arises from neuropathic mechanisms related to direct nerve damage, while in others it is driven by nociceptive sources such as muscle weakness, poor posture, and increased stress on the joints 1,2. Over time, chronic pain can interfere with everyday activities, disturb sleep, and contribute to psychological difficulties such as anxiety and depression. As the disease progresses, this burden often becomes an important physical and emotional challenge for both patients and caregivers. For this reason, pain management should be considered a central part of care, since inadequate treatment may negatively affect both overall health and quality of life [1–3].

In current clinical practice, pain management in neuromuscular diseases still relies largely on pharmacological treatment, including nonsteroidal anti-inflammatory drugs, tricyclic antidepressants, anticonvulsants such as gabapentin and pregabalin, and in refractory cases, opioids [4]. However, this approach has important limitations. Because patients with neuromuscular diseases are often vulnerable to respiratory and cardiac complications, treatment related adverse effects such as sedation, respiratory depression, and gastrointestinal intolerance may pose greater risks in this population. In addition, long term medication use may contribute to tolerance, dependence, and polypharmacy [4–6].

Physical therapy and individualized exercise programs may help prevent joint contractures, improve posture, and reduce mechanically mediated pain. Psychological interventions, particularly cognitive behavioral therapy, may also support coping with chronic pain and reduce maladaptive pain related thought patterns. In addition, complementary approaches such as acupuncture, massage therapy, and hydrotherapy may provide symptomatic relief in selected patients, but they are often insufficient to control persistent pain, particularly when neuropathic and nociceptive mechanisms coexist [7, 8].

These limitations have led to increasing interest in neuromodulation as a nonpharmacological strategy for addressing both pain and functional impairment in neuromuscular diseases [9, 10]. The aim of this review is to bring together the biological rationale and current evidence for neuromodulation in neuromuscular diseases, summarize both existing and ongoing research, and highlight potential directions for future clinical practice Table 1.

**Table 1:** Pain phenotypes across neuromuscular disorder groups

Disease group	Representative conditions	Predominant pain phenotype	Main pain drivers	Fatigue prominence	Neuromodulation relevance
Motor neuron disorders	ALS, SMA	Mixed, central, spasticity related, musculoskeletal	Immobility, spasticity, altered central excitability, secondary joint stress	High	Potentially relevant, but pain specific evidence is limited.
Peripheral neuropathies	CMT, diabetic neuropathy, radiculopathy	Predominantly neuropathic but also musculoskeletal	Peripheral nerve injury, ectopic firing, peripheral sensitization, central sensitization	Variable	Potentially relevant supported with multiple studies.
Myopathies	FSHD, DM1, DM2, LOPD	Predominantly nociceptive and mechanical	Muscle weakness, postural strain, contractures, overuse, biomechanical stress	Moderate to high	Potentially relevant but lack of evidence.

*ALS* amyotrophic lateral sclerosis, *SMA* spinal muscular atrophy, *CMT* Charcot Marie Tooth disease, *FSHD* facioscapulohumeral muscular dystrophy, *DM1* myotonic dystrophy type 1, *DM2* myotonic dystrophy type 2, *LOPD* late onset Pompe disease

## Neuromodulation: Mechanistic Basis and Supporting Evidence

Neuromodulation has emerged as a promising therapeutic approach in the management of chronic pain, based on its capacity to modulate aberrant neural activity and restore homeostatic balance within pain-processing networks [11]. Both invasive and non-invasive modalities aim to alter excitability, connectivity, and signaling in targeted circuits, thereby reducing maladaptive pain transmission [12]. This biological rationale provides the basis for understanding how neuromodulation may benefit a wide range of chronic pain conditions, extending even to those seen in neuromuscular disorders [13, 14]. The nervous system is capable of functional and structural changes in response to stimuli, a process known as neuroplasticity [15, 16]. Although neuromodulation modalities act through different mechanisms, they share functional convergence by promoting neuroplasticity [17]. This might involve both synaptic plasticity and functional modifications (e.g., ion channels) that can alter neuronal excitability. At the cellular level, stimuli can alter the electrical state of individual neurons; at the level of neurohumoral signaling, stimuli can evoke neurotransmitter activity; at the network level, stimuli can alter neuronal circuits; and at the behavioral level, stimuli can lead to changes in pain and function [18, 19]. Neuromodulation has demonstrated efficacy across a range of neuropathic pain conditions, supporting its plausibility for neuromuscular disorders. There are reported benefits of Neuromodulation for dealing with various types of painful disorders, such as, fibromyalgia, multiple sclerosis related pain, stroke pain, cancer related pain and neuropathies including both diabetic and other types [19].

Neuromuscular disorders are associated with a wide range of pain presentations, including nociceptive, neuropathic, and mixed pain components [20]. Although these disorders arise from diverse etiologies, chronic pain is a common and clinically significant problem among affected patients. In many cases, pain remains inadequately controlled with conventional pharmacological and rehabilitative approaches, highlighting an important gap in current treatment strategies [21]. The mechanistic overlap between neuropathic pain syndromes and pain in neuromuscular disorders provides a strong rationale for exploring neuromodulation in this setting [22]. Disorders such as amyotrophic lateral sclerosis, muscular dystrophies, and inherited neuropathies may involve structural and functional changes in peripheral nerves, central sensitization, and maladaptive neuroplasticity, all of which resemble mechanisms observed in conditions where neuromodulation has shown therapeutic benefit [23]. For this reason, neuromodulation may represent a promising strategy to address the persistent pain burden in neuromuscular disorders and help fill an important therapeutic gap [24].

### Transcranial Direct Current Stimulation (tDCS)

### Mechanism

Transcranial direct current stimulation is a non-invasive neuromodulation technique that delivers low-intensity constant electrical current through scalp electrodes to modulate cortical excitability. Anodal stimulation generally increases neuronal excitability, whereas cathodal stimulation reduces it, primarily through subthreshold modulation of resting membrane potentials rather than direct action potential generation [25, 26]. At the synaptic level, tDCS influences glutamatergic and GABAergic transmission and can induce long-term potentiation–like and long-term depression–like plasticity, particularly when applied repeatedly or combined with behavioral tasks [26, 27].

In the context of pain modulation, tDCS applied over the primary motor cortex or dorsolateral prefrontal cortex is thought to engage descending inhibitory pain pathways, alter thalamocortical connectivity, and reduce central sensitization [18, 27]. These mechanisms are relevant to neuromuscular disorders, where maladaptive neuroplasticity, altered sensorimotor integration, and chronic nociceptive or neuropathic inputs contribute to persistent pain. Although tDCS does not directly target peripheral pathology, its capacity to modulate central pain networks provides a mechanistic rationale for its investigation in NMD-related pain.

## Case Studies and Human Evidence

### Motor Neuron Pathologies

In amyotrophic lateral sclerosis (ALS), several human studies have evaluated tDCS, primarily focusing on safety, feasibility, and motor or neurophysiological outcomes rather than pain specific endpoints. A remotely supervised tele tDCS feasibility study demonstrated that repeated home-based stimulation was safe and well tolerated in ALS patients, with no serious adverse events reported, supporting the practicality of long-term application outside the clinic [28]. In line with these findings, earlier single subject and small cohort investigations applying anodal, cathodal, or sham motor cortex tDCS similarly reported acceptable safety profiles but failed to demonstrate consistent modulation of corticomotor excitability or disease progression, highlighting the exploratory and preliminary nature of tDCS in ALS [29].

A subsequent randomized, double blind, sham controlled trial involving 31 ALS patients reported that corticospinal tDCS significantly improved global muscle strength, caregiver burden, and quality of life scores compared with sham stimulation [30]. However, pain was not a primary outcome, and fatigue was assessed only as a secondary or exploratory measure, with no consistent or clinically meaningful improvement demonstrated. Importantly, comprehensive reviews of non-invasive brain stimulation in ALS emphasize that published tDCS studies are limited by small sample sizes, heterogeneous stimulation protocols, and outcome measures largely centered on motor function rather than symptom specific domains such as pain or fatigue [31].

From a symptom-targeted perspective, there is currently no peer-reviewed human evidence demonstrating a direct analgesic effect of tDCS in ALS. Systematic reviews of pain management in ALS emphasize that neuromodulation studies have largely prioritized disease progression and motor function, leaving pain and fatigue insufficiently explored [13]. ClinicalTrials.gov includes registered studies investigating home-based and long-term tDCS in ALS, but these protocols similarly emphasize safety and motor outcomes rather than pain-specific endpoints [32].

In spinal muscular atrophy (SMA), evidence for tDCS is even more limited. To date, there are no published clinical studies applying transcranial direct current stimulation in pediatric or adult spinal muscular atrophy populations. Nevertheless, growing neuroimaging evidence suggests that SMA is associated with altered organization of motor networks within the developing brain. In a recent resting state fMRI pilot study, children with SMA demonstrated disrupted functional connectivity within cortical motor regions together with altered cortico subcortical motor network interactions, findings that may reflect maladaptive or compensatory reorganization of motor circuits. These observations support a theoretical rationale for considering non-invasive neuromodulation approaches such as tDCS as a potential adjunct to existing therapies aimed at enhancing motor learning and network plasticity in SMA. However, this hypothesis remains speculative, as no interventional studies of tDCS in SMA have been conducted to date [33].

### Peripheral Neuropathies

While no published human studies have evaluated tDCS in Charcot–Marie–Tooth disease (CMT), robust evidence exists in other peripheral neuropathic pain conditions, particularly painful diabetic neuropathy. Multiple sham-controlled randomized trials have demonstrated that anodal tDCS applied over the primary motor cortex can significantly reduce neuropathic pain intensity in diabetic neuropathy, with reported pain reductions of approximately 30 to 40% lasting several weeks after treatment [34, 35]. These studies provide proof of concept that central neuromodulation via tDCS can meaningfully influence neuropathic pain, supporting its biological plausibility for inherited neuropathies such as CMT, despite the current absence of direct evidence.

### Inherited Myopathies

Across myopathy-dominant neuromuscular disorders, including facioscapulohumeral muscular dystrophy, myotonic dystrophy types 1 and 2, and late-onset Pompe disease, no published human studies have directly evaluated tDCS as a therapeutic intervention. A recent scoping review examining neuromodulatory interventions in muscular dystrophies have not identified transcranial direct current stimulation as an investigated modality, and available clinical literature has similarly not reported tDCS applications in these populations [14, 36]. Pain in these conditions is predominantly nociceptive or mechanical in origin, related to muscle weakness, postural strain, and contractures, which may partially explain the limited exploration of centrally acting neuromodulation strategies to date.

### Evidence Summary and Conclusion

Overall, evidence for tDCS in neuromuscular disorders remains sparse and heterogeneous. In motor neuron diseases such as ALS and SMA, existing studies demonstrate feasibility and safety but do not provide convincing evidence for pain or fatigue reduction. In myopathies, there is a complete lack of human data. In contrast, high-quality randomized evidence from painful diabetic neuropathy supports the analgesic potential of tDCS in neuropathic pain states, indirectly supporting its relevance to neuropathy-dominant NMDs.

Taken together, current data suggest that tDCS is a biologically plausible but insufficiently studied intervention for NMD-related pain. Future research should prioritize well-designed, symptom-targeted trials focusing on pain and fatigue outcomes, with careful phenotyping of NMD subgroups and standardized pain assessment tools.

## Repetitive Transcranial Magnetic Stimulation (rTMS)

### Mechanism

Repetitive transcranial magnetic stimulation is a non-invasive neuromodulation technique that uses rapidly changing magnetic fields to induce electric currents in cortical tissue, thereby modulating neuronal excitability and synaptic plasticity. Depending on stimulation frequency, pattern, and cortical target, rTMS can produce either facilitatory or inhibitory effects on cortical networks. High-frequency stimulation (≥ 5 Hz) and intermittent theta burst stimulation generally increase cortical excitability, whereas low-frequency stimulation (≤ 1 Hz) and continuous theta burst stimulation tend to suppress excitability [37, 38].

From a pain-modulation perspective, rTMS applied over the primary motor cortex is believed to activate descending inhibitory pain pathways, modulate thalamocortical dysrhythmia, and normalize maladaptive cortical reorganization associated with chronic pain states [39]. Neurochemical studies suggest rTMS influences glutamatergic transmission, GABAergic inhibition, endogenous opioid release, and monoaminergic systems, all of which are implicated in central pain processing [40]. These mechanisms overlap substantially with pathophysiological features observed in neuromuscular disorders, including altered sensorimotor integration, central sensitization, and impaired cortical inhibition.

## Case Evidence and Human Studies

### Motor Neuron Disorders

In ALS, the majority of rTMS studies have focused on disease progression, cortical excitability, and motor outcomes rather than symptom-directed analgesia. An early double blind, placebo controlled study investigated continuous theta burst stimulation of the motor cortex and reported a modest but significant slowing of ALS functional decline over six months, although the small sample size limited definitive conclusions [41]. Importantly, pain was not a primary outcome.

Fatigue has been evaluated in a limited number of ALS rTMS studies. Zanette et al. conducted a controlled study using 5 Hz rTMS in ALS patients and included fatigue and quality-of-life measures among secondary outcomes, making it one of the few ALS rTMS datasets to formally assess fatigue [42]. While the intervention was well tolerated, fatigue outcomes showed only modest and inconsistent changes, reflecting the exploratory nature of the evidence. Subsequent reviews of fatigue management in ALS emphasize that interventional data remain sparse and methodologically heterogeneous, with rTMS evidence classified as low certainty [43].

With respect to pain, no peer-reviewed rTMS case reports or trials in ALS have specifically targeted analgesia or reported clinically meaningful pain reduction. Scoping reviews and PubMed-indexed searches consistently indicate an absence of symptom-directed rTMS analgesia studies in ALS, highlighting a significant evidence gap [13, 43].

In SMA, rTMS has predominantly been used as a neurophysiological assessment tool rather than a therapeutic intervention. Existing studies employ single-pulse or paired-pulse TMS to evaluate corticospinal tract integrity and cortical excitability, without repetitive stimulation protocols or clinical outcome reporting. To date, therapeutic rTMS in SMA is identifiable only at the level of clinical trial registration. The ongoing study evaluating safety and tolerability of low motoneuron stimulation via rTMS in SMA (NCT06977269) focuses on safety and motor outcomes, and no peer-reviewed results or pain-related data have been published [44]. This underscores the absence of clinical evidence supporting rTMS for pain or fatigue in SMA.

### Peripheral Neuropathies

Robust analgesic effects of rTMS have been demonstrated in non-NMD neuropathic pain conditions, including diabetic neuropathy, post-stroke pain, and spinal cord injury–related pain, where high-frequency motor cortex rTMS has produced clinically significant pain reduction in randomized controlled trials [39, 45]. These data provide indirect support for the potential applicability of rTMS in neuropathy-dominant neuromuscular disorders, despite the current lack of direct evidence.

### Inherited Myopathies

There are no published rTMS studies targeting pain in inherited peripheral neuropathies such as CMT disease or in myopathy-dominant neuromuscular disorders including facioscapulohumeral muscular dystrophy, myotonic dystrophy, or late-onset Pompe disease. In these conditions, rTMS has either not been studied at all or has been limited to neurophysiological investigations without therapeutic intent [14, 36].

### Evidence Summary and Potential Applicability

Overall, evidence for rTMS in neuromuscular disorders is limited and largely indirect. In ALS, small, controlled studies demonstrate safety and modest effects on cortical excitability and disease progression, with fatigue occasionally assessed but pain rarely, if ever, evaluated as a primary outcome. In SMA and myopathy-dominant disorders, therapeutic rTMS remains largely unexplored, with evidence confined to neurophysiological assessments or trial registrations without published results.

Despite these limitations, the established efficacy of rTMS for neuropathic pain in other neurological conditions, combined with shared mechanisms such as central sensitization and maladaptive cortical plasticity, supports rTMS as a biologically plausible but underinvestigated intervention for NMD-related pain. Future research should prioritize symptom-targeted rTMS trials with standardized pain and fatigue outcomes, stratified by NMD pathology type.

## Vagal Nerve Stimulation (VNS)

### Mechanism

Vagal nerve stimulation is a neuromodulation technique that modulates afferent and efferent vagal pathways projecting to key brainstem and supraspinal structures involved in pain processing, including the nucleus tractus solitarius, locus coeruleus, dorsal raphe nucleus, thalamus, and limbic regions. Through these connections, VNS influences noradrenergic and serotonergic neurotransmission and enhances descending inhibitory pain pathways [46, 47]. Additionally, VNS exerts anti-inflammatory effects via the cholinergic anti-inflammatory reflex, reducing proinflammatory cytokine release such as tumor necrosis factor alpha and interleukin 6, which are increasingly recognized as contributors to chronic pain states [48].

Both invasive cervical VNS and non-invasive transcutaneous auricular VNS aim to engage similar central mechanisms, although with differing stimulation intensities and neural recruitment patterns. Functional neuroimaging studies demonstrate that VNS alters activity in pain related cortical and subcortical networks, including the insula, anterior cingulate cortex, and prefrontal regions, supporting its mechanistic relevance to chronic pain modulation [49].

## Case Evidence and Human Studies

### Motor Neuron Disorders

In amyotrophic lateral sclerosis, human evidence for VNS remains extremely limited. No peer reviewed randomized or controlled trials evaluating invasive or transcutaneous VNS for pain or fatigue in ALS have been published. Available literature consists primarily of preclinical studies and theoretical frameworks suggesting that vagal modulation may attenuate neuroinflammation and slow neurodegeneration [50]. Small unpublished or non-indexed pilot experiences have been referenced in narrative reviews, but these reports lack sufficient methodological detail and are not suitable for evidence-based conclusions.

From a symptom focused perspective, there is currently no clinical evidence supporting VNS as an intervention for pain or fatigue in ALS. Systematic reviews of pain management strategies in ALS consistently note the absence of vagal neuromodulation trials targeting pain outcomes [13]. Similarly, ClinicalTrials.gov searches do not identify any registered trials evaluating VNS specifically for pain or fatigue in ALS populations.

In spinal muscular atrophy, no published human studies or registered clinical trials have investigated VNS as a therapeutic modality. The absence of data likely reflects both the rarity of the condition and the prioritization of disease modifying therapies and motor focused interventions over symptom targeted neuromodulation.

### Peripheral Neuropathies

Although direct evidence in inherited neuromuscular disorders is lacking, emerging data from peripheral neuropathic pain conditions provide indirect support for the analgesic potential of VNS. Small pilot studies of transcutaneous auricular VNS in patients with diabetic peripheral neuropathy have demonstrated modest reductions in pain intensity and improvements in autonomic markers, suggesting engagement of pain modulatory circuits while a study shows it has no anti-inflammatory effect in diabetes [51, 52]. More recently, exploratory studies combining vagal and cranial nerve stimulation approaches have reported substantial pain reductions in severe diabetic neuropathy, although these findings are derived from uncontrolled cohorts and should be interpreted with caution [53].

### Inherited Myopathies

In myopathy dominant neuromuscular disorders such as facioscapulohumeral muscular dystrophy, myotonic dystrophy, and late onset Pompe disease, no human studies of VNS have been reported. Pain in these conditions is predominantly nociceptive or mechanical, and the role of centrally mediated neuromodulation strategies such as VNS remains speculative.

### Evidence Summary and Potential Applicability

Current evidence does not support the clinical use of VNS for pain or fatigue management in neuromuscular disorders [52]. However, mechanistic overlap between neuroinflammation, autonomic dysregulation, and chronic pain provides a biologically plausible rationale for further investigation.

Indirect evidence from diabetic neuropathy suggests that noninvasive VNS may modulate pain processing pathways and autonomic balance, indicating potential applicability to neuropathy dominant neuromuscular disorders. Future research should focus on well-designed pilot trials assessing safety, feasibility, and pain specific outcomes of transcutaneous VNS in selected NMD populations before considering larger efficacy trials.

## Spinal Cord Stimulation (SCS)

### Mechanism

Spinal cord stimulation is an invasive neuromodulation technique involving epidural delivery of electrical stimulation to the dorsal columns of the spinal cord. SCS modulates nociceptive transmission primarily through activation of large diameter Aβ fibers, resulting in segmental inhibition of pain signaling at the dorsal horn level, consistent with gate control theory [54]. Beyond segmental effects, SCS also influences supraspinal pain networks by altering thalamic activity, engaging descending inhibitory pathways, and modulating neurotransmitter systems including gamma aminobutyric acid, serotonin, and norepinephrine [55].

Modern SCS paradigms extend beyond traditional tonic stimulation to include high frequency (10 kHz), burst, and closed loop stimulation, each producing analgesia through partially distinct mechanisms. High frequency SCS appears to suppress dorsal horn hyperexcitability without paresthesia, while burst stimulation may preferentially target affective components of pain via medial pain pathways [56, 57]. These mechanisms are particularly relevant to neuromuscular disorders characterized by chronic neuropathic pain, central sensitization, and altered spinal excitability.

## Case Evidence and Human Studies

### Motor Neuron Pathologies

In motor neuron diseases such as amyotrophic lateral sclerosis, evidence for SCS is extremely sparse. Isolated case reports describe use of cervical or thoracic SCS for refractory musculoskeletal or neuropathic pain in ALS patients, with reported improvement in pain intensity but no impact on disease progression or motor decline [58]. No controlled studies or randomized trials have evaluated SCS in ALS, and pain remains a secondary or incidental indication rather than a primary therapeutic target.

In SMA, recent first in human studies have investigated epidural SCS as a strategy to enhance motor function rather than pain relief. Pilot studies in adults with SMA type 3 demonstrated improvements in muscle strength, endurance, and gait following lumbosacral epidural stimulation, with fatigue related endurance measures showing improvement, but pain outcomes were not systematically assessed [59, 60]. These findings suggest that while SCS may modulate spinal motor circuits in SMA, its role in pain management remains undefined.

### Peripheral Neuropathies

Among neuromuscular disorders, the strongest evidence base for SCS exists in peripheral neuropathy dominant conditions. In painful diabetic neuropathy, multiple high quality randomized controlled trials have demonstrated robust and sustained analgesic efficacy of SCS. A trial showed that 10 kHz SCS combined with standard medical therapy resulted in significant pain reduction in over 80% of patients, compared with minimal improvement in the medical therapy alone group, with benefits sustained at long term follow up ^61^. These findings have established SCS as an evidence-based treatment option for refractory diabetic neuropathic pain. In inherited neuropathies, evidence is more limited but suggestive. A published case report described successful long term pain control with SCS in a patient with Charcot–Marie–Tooth disease suffering from severe neuropathic pain refractory to conservative treatment [62]. Although limited to individual cases, these reports indicate potential applicability of SCS to genetically mediated neuropathic pain states.

SCS has also demonstrated efficacy in other neuropathic pain conditions relevant to neuromuscular populations, including radiculopathy and peripheral nerve injury. Large systematic review and guideline statements support SCS use in these conditions, providing indirect support for its consideration in neuropathy dominant neuromuscular disorders [63].

### Inherited Myopathies

In myopathy dominant neuromuscular disorders including facioscapulohumeral muscular dystrophy, myotonic dystrophy, and late onset Pompe disease, no published human studies have evaluated SCS for pain or other symptoms. Pain in these conditions is predominantly nociceptive or mechanical, and invasive spinal neuromodulation has not been explored.

### Evidence Summary and Potential Applicability

Spinal cord stimulation represents the neuromodulation modality with the strongest clinical evidence for neuropathic pain among conditions overlapping with neuromuscular disorders. Robust randomized data support its use in painful diabetic neuropathy and radiculopathy, and limited case evidence suggests potential benefit in inherited neuropathies such as Charcot–Marie–Tooth disease.

In contrast, evidence for SCS in motor neuron and myopathy dominant neuromuscular disorders is minimal and largely exploratory. Existing studies in SMA focus on motor enhancement rather than pain, while ALS data are confined to isolated case reports. Consequently, SCS should currently be considered a viable option primarily for neuropathy dominant neuromuscular disorders with refractory neuropathic pain, rather than as a general pain management strategy across all NMDs.

Future research should include carefully selected neuromuscular subpopulations with neuropathic pain phenotypes, standardized pain outcomes, and modern SCS paradigms to clarify the therapeutic role of SCS in this heterogeneous group.

## Dorsal Root Ganglion (DRG) Stimulation

### Mechanism

Dorsal root ganglion stimulation is an invasive neuromodulation technique that delivers targeted electrical stimulation directly to the dorsal root ganglia, structures that contain the cell bodies of primary sensory neurons. By modulating afferent nociceptive signaling at this anatomically strategic site, DRG stimulation allows precise, dermatomally specific pain control with reduced stimulation of non-involved regions compared with conventional spinal cord stimulation [64, 65].

Mechanistically, DRG stimulation is thought to suppress ectopic discharges from injured sensory neurons, stabilize hyperexcitable membrane potentials, and reduce abnormal spontaneous firing associated with neuropathic pain. Preclinical and human neurophysiological studies suggest that DRG stimulation modulates sodium and calcium channel activity, attenuates central sensitization at the dorsal horn level, and alters ascending pain transmission without relying on broad dorsal column engagement [66, 67]. These properties make DRG stimulation particularly suitable for focal and distal neuropathic pain phenotypes.

## Case Evidence and Human Studies

### Motor Neuron Pathologies

To date, no published human studies have investigated DRG stimulation in motor neuron diseases such as amyotrophic lateral sclerosis or spinal muscular atrophy. Pain in these disorders is typically multifactorial, involving spasticity, immobility related musculoskeletal pain, and mixed central mechanisms, which may limit the applicability of focal sensory neuromodulation approaches.

### Peripheral Neuropathies

The strongest clinical evidence for DRG stimulation exists in peripheral neuropathic pain conditions. Multiple prospective studies and case series have demonstrated significant and sustained pain reduction with DRG stimulation in patients with complex regional pain syndrome, post-surgical neuropathic pain, and focal radiculopathies [68–70]. Additionally, experimental studies have demonstrated a significant reduction in pain intensity in animal models of painful diabetic neuropathy following DRG stimulation [71–73]. These findings have established DRG stimulation as an effective option for anatomically localized neuropathic pain refractory to conventional therapies.

In painful diabetic neuropathy, emerging evidence supports the efficacy of DRG stimulation. Retrospective and prospective cohort studies report mean pain reductions exceeding 60% at 6 to 12 months, with improvements in quality of life comparable to those achieved with high frequency spinal cord stimulation [70, 74]. Although randomized controlled trials remain limited, these data suggest that DRG stimulation may represent a viable alternative for patients with distal symmetric neuropathic pain who do not respond to conventional SCS [75].

In inherited neuropathies such as Charcot–Marie–Tooth disease, no published case reports or clinical trials have specifically evaluated DRG stimulation. However, given the focal and length dependent neuropathic pain patterns observed in some CMT subtypes, DRG stimulation is theoretically well suited to this population, although this remains speculative in the absence of human data.

### Inherited Myopathies

Similarly, no evidence exists for DRG stimulation in myopathy dominant neuromuscular disorders including facioscapulohumeral muscular dystrophy, myotonic dystrophy, or late onset Pompe disease. Pain in these conditions is predominantly nociceptive or mechanical, and DRG stimulation has not been explored.

### Evidence Summary and Potential Applicability

Dorsal root ganglion stimulation is a well-supported neuromodulation strategy for focal neuropathic pain conditions, with growing evidence in painful diabetic neuropathy and radiculopathy. Its ability to provide dermatomally precise analgesia distinguishes it from traditional spinal cord stimulation and may offer advantages in selected neuropathy dominant neuromuscular disorders [75].

However, direct evidence for DRG stimulation in inherited neuromuscular disorders is currently absent. No human data exist in motor neuron or myopathy dominant NMDs, and applicability in inherited neuropathies such as CMT remains theoretical. At present, DRG stimulation should be considered only in carefully selected patients with neuromuscular disorders who exhibit focal neuropathic pain phenotypes analogous to established DRG indications.

Future research priorities include prospective registries and pilot trials evaluating DRG stimulation in genetically mediated neuropathies, with standardized pain outcomes and long term follow up.

## Peripheral Nerve Stimulation (PNS)

### Mechanism

Peripheral nerve stimulation is a neuromodulation technique that delivers electrical or magnetic stimulation directly to peripheral nerves or their terminal branches in order to modulate nociceptive transmission before signals enter the central nervous system. PNS primarily acts by activating large diameter Aβ fibers, inhibiting nociceptive Aδ and C fiber activity at the spinal dorsal horn level, and reducing ectopic discharges arising from injured peripheral nerves [76, 77]. In addition, PNS can induce local and central neuroplastic changes, including normalization of cortical somatotopic representations and modulation of descending inhibitory pathways [78].

Compared with spinal cord and dorsal root ganglion stimulation, PNS provides a more anatomically distal and focal intervention, making it particularly suitable for localized neuropathic pain syndromes. Contemporary PNS systems include temporary percutaneous leads, fully implanted systems, and non-invasive magnetic or transcutaneous approaches, each engaging similar biological mechanisms with differing invasiveness profiles [22, 79].

## Case Evidence and Human Studies

### Motor Neuron Pathologies

In motor neuron diseases, the role of PNS is limited and context dependent. In amyotrophic lateral sclerosis, peripheral nerve stimulation has primarily been explored in the form of diaphragmatic or phrenic nerve pacing aimed at respiratory support rather than pain management. Randomized trials demonstrated no benefit and potential harm with diaphragmatic pacing in ALS, and pain outcomes were not targeted [80, 81]. Consequently, PNS cannot currently be recommended for pain management in ALS.

In spinal muscular atrophy, peripheral nerve stimulation has been reported only in isolated historical cases, such as phrenic nerve pacing for ventilatory support, without assessment of pain or fatigue outcomes [82]. No contemporary trials have evaluated PNS for pain relief in SMA.

### Peripheral Neuropathies

The strongest evidence base for PNS in neuromuscular relevant conditions exists in peripheral neuropathic pain syndromes. In painful diabetic neuropathy, recent randomized controlled trials evaluating both implanted and non-invasive magnetic PNS have demonstrated clinically meaningful pain reduction. A double-blind randomized trial of magnetic peripheral nerve stimulation reported that over 70% of treated patients achieved at least 50% pain reduction, with sustained benefit over several months [83, 84]. These findings position PNS as a promising option for refractory diabetic neuropathic pain.

In inherited peripheral neuropathies such as Charcot–Marie–Tooth disease, evidence remains limited. A historical case report described improvement in muscle function following transcutaneous peripheral nerve stimulation in a CMT patient, although pain was not the primary outcome [85]. More recently, a randomized clinical trial evaluating transcutaneous electrical nerve stimulation for pain relief in CMT1A patients has been registered, indicating growing interest in peripheral neuromodulation for this population, although peer reviewed results are pending [86].

PNS has also demonstrated efficacy in traumatic nerve injury and focal neuropathic pain states, including post-surgical neuropathy and radiculopathy. Systematic reviews and consensus statements recognize PNS as an effective treatment for well localized neuropathic pain, supporting its translational relevance to neuropathy dominant neuromuscular disorders [79, 87].

### Inherited Myopathies

In myopathy dominant neuromuscular disorders including facioscapulohumeral muscular dystrophy, myotonic dystrophy, and late onset Pompe disease, PNS has not been systematically studied for pain management. Pain in these conditions is largely nociceptive or mechanical, and peripheral neuromodulation strategies have not been explored beyond supportive or rehabilitative contexts.

### Evidence Summary and Potential Applicability

Peripheral nerve stimulation is a well-supported intervention for localized neuropathic pain conditions and has expanding evidence base in painful diabetic neuropathy and focal nerve injuries. Its minimally invasive nature and anatomical specificity make it an attractive option for neuropathy dominant neuromuscular disorders.

However, direct evidence supporting PNS in inherited neuromuscular diseases remains sparse. In motor neuron and myopathy dominant disorders, existing applications are limited to respiratory support rather than pain control, with no evidence of analgesic benefit. At present, PNS should be considered primarily for neuromuscular patients with focal neuropathic pain phenotypes that closely resemble established PNS indications.

Future research should focus on prospective trials evaluating PNS for pain in inherited neuropathies such as CMT, with standardized pain outcomes and careful phenotypic stratification.

## Trigeminal Nerve Stimulation (TNS)

### Mechanism

Trigeminal nerve stimulation is a non-invasive neuromodulation technique that targets sensory branches of the trigeminal nerve, most commonly the supraorbital and supratrochlear branches, using transcutaneous electrical stimulation. Afferent trigeminal fibers project to the trigeminal sensory nuclei in the brainstem, which have extensive connections with pain modulatory regions including the locus coeruleus, periaqueductal gray, thalamus, anterior cingulate cortex, and insular cortex [88, 89]. Through these pathways, TNS can influence both sensory discriminative and affective motivational components of pain.

TNS modulates monoaminergic neurotransmission, enhances descending inhibitory pain control, and alters thalamocortical excitability. Neuroimaging data demonstrate that trigeminal stimulation induces changes in functional connectivity within pain related cortical networks, supporting its mechanistic relevance to chronic pain modulation [89].

## Case Evidence and Human Studies

### Motor Neuron Disorders (ALS and SMA)

In motor neuron diseases, evidence for TNS is extremely limited. No peer reviewed clinical trials or case reports have evaluated trigeminal nerve stimulation for pain or fatigue management in amyotrophic lateral sclerosis. Existing literature on neuromodulation in ALS focuses predominantly on cortical or spinal targets, and trigeminal pathways have not been explored in this population [13].

Similarly, in spinal muscular atrophy, no human studies or registered clinical trials have investigated TNS. Given that pain in SMA is often secondary to postural abnormalities, joint stress, and muscle weakness rather than focal cranial sensory dysfunction, the lack of investigation into trigeminal neuromodulation is not unexpected.

### Peripheral Neuropathies and Other Neuromuscular Disorders

While direct evidence in neuromuscular disorders is absent, TNS has been investigated in other neurological pain conditions. Randomized controlled trials and open label studies have demonstrated analgesic and preventive effects of external trigeminal nerve stimulation in migraine and cluster headache, supporting its capacity to modulate central pain processing networks [90, 91]. These findings provide indirect support for potential applicability of TNS to neuropathic or centrally mediated pain states.

In diabetic neuropathy, an exploratory observational study combining auricular vagus nerve stimulation with trigeminal or trigeminocervical stimulation reported substantial reductions in neuropathic pain intensity. However, the study lacked a control group and involved multimodal stimulation, precluding attribution of effects specifically to TNS [53]. No standalone TNS trials have been conducted in diabetic neuropathy or inherited peripheral neuropathies such as Charcot–Marie–Tooth disease.

### Inherited Myopathies

In myopathy dominant neuromuscular disorders including facioscapulohumeral muscular dystrophy, myotonic dystrophy, and late onset Pompe disease, no studies have examined TNS. Pain mechanisms in these conditions are predominantly nociceptive or mechanical, and trigeminal pathways are unlikely to represent primary therapeutic targets.

### Evidence Summary and Potential Applicability

At present, there is no direct clinical evidence supporting the use of trigeminal nerve stimulation for pain or fatigue management in neuromuscular disorders. Human data in ALS, SMA, inherited neuropathies, and myopathies are entirely absent.

Nevertheless, established efficacy of TNS in primary headache disorders and preliminary signals from exploratory neuropathic pain studies suggest that trigeminal modulation can influence central pain networks. This positions TNS as a biologically plausible but highly experimental approach for NMD related pain, particularly in conditions with prominent central sensitization.

**Fig. 1:**
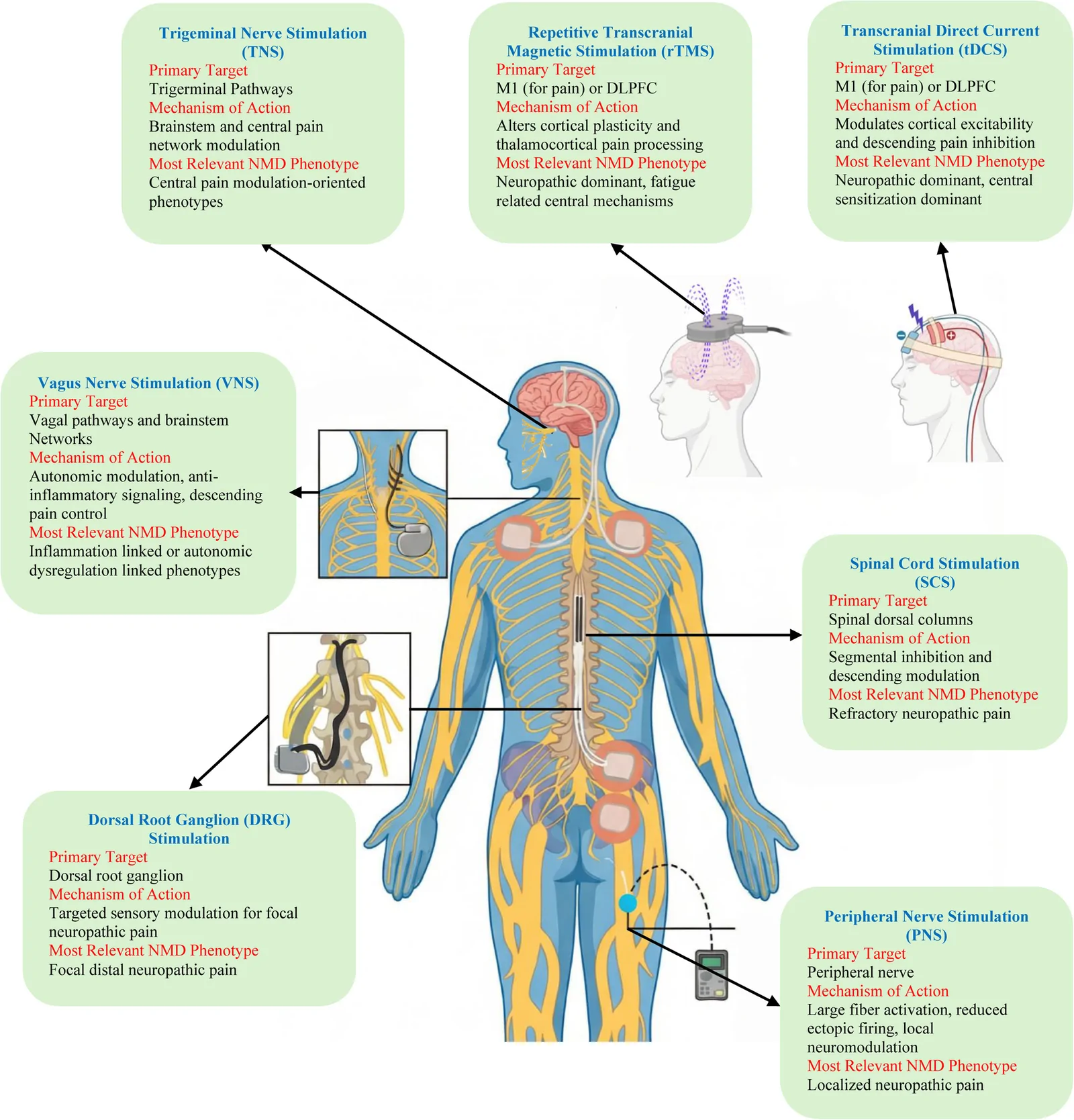
Overview of neuromodulation modalities, targets, and proposed mechanisms

Future research, if pursued, should begin with small feasibility studies using noninvasive TNS in carefully selected neuromuscular populations, with rigorous pain phenotyping and conservative expectations regarding efficacy Fig. 1, Table 2 and 3.

**Table 2:** Comparison of neuromodulation modalities in neuromuscular pain and fatigue

Modality	Type	Primary target	Proposed mechanism	Most relevant NMD phenotype
tDCS	Noninvasive	Cortex (M1)	Modulates cortical excitability and descending pain inhibition	Neuropathic dominant, central sensitization dominant
rTMS	Noninvasive	Cortex	Alters cortical plasticity and thalamocortical pain processing	Neuropathic dominant, fatigue related central mechanisms
VNS	Invasive or noninvasive	Vagal pathways and brainstem networks	Autonomic modulation, anti-inflammatory signaling, descending pain control	Inflammation linked or autonomic dysregulation linked phenotypes
SCS	Invasive	Spinal dorsal columns	Segmental inhibition and descending modulation	Refractory neuropathic pain
DRG stimulation	Invasive	Dorsal root ganglion	Targeted sensory modulation for focal neuropathic pain	Focal distal neuropathic pain
PNS	Invasive or noninvasive	Peripheral nerve	Large fiber activation, reduced ectopic firing, local neuromodulation	Localized neuropathic pain
TNS	Noninvasive	Trigeminal pathways	Brainstem and central pain network modulation	Central pain modulation-oriented phenotypes

*tDCS* transcranial direct current stimulation, *rTMS* repetitive transcranial magnetic stimulation, *VNS* vagus nerve stimulation, *SCS* spinal cord stimulation, *DRG* dorsal root ganglion, *PNS* peripheral nerve stimulation, *TNS* trigeminal nerve stimulation, *NMD* neuromuscular disorder, *M1* primary motor cortex.

**Table 3:** Disease by modality evidence matrix

Disease group	Representative conditions	tDCS	rTMS	VNS	SCS	DRG Stimulation	PNS	TNS
Motor neuron disorders	ALS	Feasibility and safety demonstrated, but no convincing evidence for pain or fatigue reduction^28–32^.	Studies reported slowing of functional decline but did not evaluate pain relief^41^. A few studies assessed fatigue as a secondary outcome^42^.	N/R	Limited to isolated case reports showing pain improvement without effects on progression or motor decline^58^. No controlled studies are available, and pain has remained a secondary indication.	N/R	Primarily studied for respiratory support rather than pain, with trials showing no benefit and possible harm^80,81^.	N/R
	SMA	N/R	An ongoing trial assesses safety and tolerability, with no published pain related findings^44^.	N/R	Pilot studies showed improvements in strength, endurance, and gait, but pain outcomes were not systematically assessed. Fatigue related endurance measures also improved^59,60^.	N/R	Reported only in isolated historical cases for ventilatory support, without pain or fatigue assessment^82^.	N/R
Peripheral neuropathies	CMT and inherited neuropathies	N/R	N/R	N/R	A case report described successful long term pain control in refractory severe neuropathic pain^62^.	N/R	A historical case report noted functional improvement^85^, while a registered trial is evaluating pain relief with results pending^86^.	N/R
	Painful diabetic neuropathy as comparator	Sham controlled trials showed that anodal M1 tDCS reduced neuropathic pain in diabetic neuropathy by about 30% to 40%, with effects lasting for weeks^34,35^.	Robust analgesic effects have been shown in non NMD neuropathic pain conditions, with clinically significant pain reduction in randomized trials^39,45^.	Pilot studies showed modest pain and autonomic improvement in diabetic neuropathy. Combined vagal and cranial stimulation showed greater pain relief, but evidence is preliminary^51–53^	A RCT in painful diabetic neuropathy showed that 10 kHz stimulation achieved pain reduction in over 80% of patients, with effects sustained long term^61^.	Prospective studies showed significant and sustained pain reduction in localized peripheral neuropathic pain^68–70^. In painful diabetic neuropathy, cohort studies reported over 60% pain reduction at 6 to 12 months^70,74^.	RCTs showed clinically meaningful pain reduction in painful diabetic neuropathy. In a double-blind trial, over 70% achieved at least 50% pain reduction with sustained effects over months^83,84^.	An exploratory observational study reported substantial pain reduction^53^.
Myopathies	FSHD, DM1, DM2, LOPD	N/R	N/R	N/R	N/R	N/R	N/R	N/R

*ALS* amyotrophic lateral sclerosis, *SMA* spinal muscular atrophy, *CMT* Charcot Marie Tooth disease, *FSHD* facioscapulohumeral muscular dystrophy, *DM1* myotonic dystrophy type 1, *DM2* myotonic dystrophy type 2, *LOPD* late onset Pompe disease, *tDCS* transcranial direct current stimulation, *rTMS* repetitive transcranial magnetic stimulation, *VNS* vagus nerve stimulation, *SCS* spinal cord stimulation, *DRG* dorsal root ganglion stimulation, *PNS* peripheral nerve stimulation, *TNS* trigeminal nerve stimulation, *RCT* randomized controlled trial, *N/R* not reported

## Limitations of Current Evidence

Current evidence suggests that neuromodulation may be a promising option for pain and fatigue in neuromuscular disorders, especially when symptoms seem to reflect neuropathic pain mechanisms, altered cortical excitability, or broader changes in brain plasticity [10, 20, 92].

Still, the clinical evidence remains limited and should be interpreted cautiously, because most of the available literature is exploratory rather than definitive [67, 71]. Across neuromuscular disorders, the published evidence is dominated by reviews, case-based observations, small pilot studies, and early feasibility trials, with only limited controlled data in selected conditions such as idiopathic inflammatory myopathies [20, 93]. As a result, the rationale for neuromodulation in this population still depends heavily on mechanistic overlap and evidence drawn from the broader neuropathic pain literature rather than on strong disease specific trials [24]. One of the clearest limitations of the current literature is the lack of well-designed randomized and sham controlled studies, especially crossover trials that treat pain as a primary outcome. Apart from a small number of exceptions, such as the randomized, double blind, sham controlled crossover trial in idiopathic inflammatory myopathies, most studies in neuromuscular disorders still rely on early stage or exploratory designs rather than robust pain focused trials [93]. This gap is particularly evident in ALS, where many neuromodulation studies have focused mainly on safety, feasibility, cortical excitability, or disease progression instead of pain relief itself. In practice, the field has been driven more by neurophysiologic rationale and proof of concept work than by trials designed around pain outcomes [28, 29, 32, 41]. For invasive approaches such as spinal cord stimulation, dorsal root ganglion stimulation, and peripheral nerve stimulation, the evidence is even more limited in inherited neuropathies and other neuromuscular populations. Direct evidence within these groups is still largely restricted to isolated reports, such as the Charcot Marie Tooth spinal cord stimulation case, while much of the broader support comes from painful diabetic neuropathy cohorts or from the wider peripheral neuropathic pain literature rather than from disease specific neuromuscular studies [62, 70, 74, 94]. Another major challenge is the marked heterogeneity of neuromuscular disorders themselves. Across this broad group, pain and fatigue do not arise from a single mechanism or follow a uniform clinical pattern. Instead, patients may present with nociceptive, neuropathic, mixed, or fatigue dominant symptom profiles, and even within the same disease category the severity, distribution, and functional impact of these symptoms can vary substantially. This makes it difficult to compare studies directly or to generalize findings from one neuromuscular population to another [1, 2, 14, 20, 95]. The field is also weakened by methodological inconsistency. Available neuromodulation studies often include small and heterogeneous samples, use different stimulation targets and treatment parameters, and rely on outcome measures that are not aligned across studies. In addition, pain and fatigue are not assessed with standardized, disease appropriate tools in a consistent way, and several study programs still emphasize strength, balance, or functional outcomes more than symptom specific endpoints [23, 36, 96].

## Future Directions

Future research should prioritize larger, methodologically rigorous multicenter trials with clearer phenotypic stratification and disease specific eligibility criteria, since broad neuromuscular labels often obscure important differences in pain phenotype, fatigue burden, and underlying mechanisms [20, 93]. Enrichment strategies based on neuropathic pain features, altered cortical plasticity, neurophysiologic signatures, and relevant biomarkers may help identify patients most likely to benefit and improve the mechanistic interpretation of treatment response [92, 97]. Future studies should also evaluate neuromodulation within multimodal care pathways, particularly alongside rehabilitation and, when appropriate, pharmacologic therapy, while adopting standardized pain, fatigue, and patient reported outcome measures tailored to neuromuscular populations to improve comparability across studies and support clinical translation [24, 98].

## Conclusions

Pain and fatigue remain major and often insufficiently managed problems in neuromuscular disorders. Current evidence suggests that neuromodulation may represent a promising therapeutic option for selected patients, particularly when conventional pharmacologic approaches provide limited benefit or fail to address the multidimensional nature of symptoms. However, the available literature is still heterogeneous, preliminary, and largely based on small studies or early clinical experience. At present, neuromodulation cannot be considered a standard treatment across neuromuscular conditions. More randomized controlled trials, sham controlled crossover studies, multicenter prospective cohorts, and carefully characterized case series are needed to clarify efficacy, refine patient selection, standardize outcome measures, and better define the role of neuromodulation in clinical practice.

## Data Availability

No datasets were generated or analysed during the current study.
